# Supplementary material for: Development of Loop-Mediated Isothermal Amplification (LAMP) Assays for the Rapid Detection of Toxigenic Aspergillus flavus and A. carbonarius in Nuts
Source: Int J Mol Sci. 2024 Mar 29;25(7):3809. doi: 10.3390/ijms25073809 (PMC11011790; doi:10.3390/ijms25073809)
Supplement: Supplementary file 1 [file ijms-25-03809-s001.zip › ijms-2911869-supplementary.pdf]

Supplementary data

Development of Loop-Mediated Isothermal Amplification (LAMP) Assays for the Rapid Detection of Toxigenic *Aspergillus flavus* and *A. carbonarius* in Nuts

Wanissa Mellikeche [1], Alessandra Ricelli [2,*], Giulia Casini [3], Marilita Gallo [4], Nuray Baser [4], Giancarlo Colelli [1] and Anna Maria D'Onghia [4]

[1] Department of Agricultural Sciences, Food, Natural Resources and Engineering, University of Foggia, Via Napoli, 25-71122 Foggia, Italy; wanissa.mellikeche@unifg.it (W.M.); giancarlo.colelli@unifg.it (G.C.)
[2] National Research Council—Institute of Molecular Biology and Pathology, P.le A. Moro, 5-00185 Rome, Italy
[3] Enbiotech SRL, Via Del Bersagliere, 45-90143 Palermo, Italy; g.casini@enbiotech.eu
[4] International Centre for Advanced Mediterranean Agronomic Studies, Via Ceglie, 9-70010 Valenzano, Italy; gallo@iamb.it (M.G.); baser@iamb.it (N.B.); donghia@iamb.it (A.M.D.)
* Correspondence: alessandra.ricelli@cnr.it

Table 1. Fungal strains used for the development, optimization and validation of LAMP assays for the detection A. flavus and A. carbonarius

A. carbonarius	BP36<br>A129<br>A135<br>A143<br>A144<br>A150<br>A156<br>A157<br>A161<br>A168
A. flavus	BP50<br>BP53<br>AS6<br>AS3<br>AS9<br>AS15<br>AS10<br>AS8<br>AS7<br>ACa10
A. niger	BP4<br>BP8<br>BP10<br>BP16<br>BP18<br>BP26<br>BP27<br>BP30<br>BP31<br>BP34<br>BP35<br>BP37

	BP51<br>BP55<br>AS2<br>AS22<br>ACa1
A. tubingensis	BP29<br>BP33<br>BP41<br>BP43
A. tamarii	AS17
A. parasiticus	A.P1
Penicillium spp.	A1P1<br>A1P5<br>A1P6<br>A1P8<br>A1P13

Table 2 Sensitivity parameters obtained with primer sets ACS02 and AFS03 on pistachio and almond nuts

	**Set/Matrix combination**			
Parameters	ACS02/Pistachio	ACS02/Almond	AFS03/Pistachio	AFS03/Almond
Sensitivity of an alternative method	100%	100%	100%	100%
Sensitivity of the reference method	100%	100%	100%	100%
Relative accuracy	100%	100%	100%	100%
Ratio of false positive results for the alternative method	0%	0%	0%	0%

Table 3 RLOD test results for the primer set ACS02 on pistachio and almond nuts

Pistachio samples	Alternative method	Reference method
Non-inoculated	0%	0
Inoculated 100 spores	100%	100%
Inoculated 10 spores	100%	100%
Almond samples	Alternative method	Reference method
Non-inoculated	0%	0
Inoculated 100 spore	80%	100%
Inoculated 10 spores	100%	100%

Table 4 RLOD test results for the primer set AFS03 on pistachio and almond nuts

Pistachio samples	Alternative method	Reference method
Non-inoculated	0%	0
Inoculated 100 spore	80%	100%
Inoculated 10 spores	100%	100%
Almond samples	Alternative method	Reference method
Non-inoculated	0	0
Inoculated 100 spore	100%	100%
Inoculated 10 spores	100%	100%

Table 5 Inclusivity test results for the primer set ACS02

Strain	Species	Result
BP36	*A. carbonarius*	+
A.C1	*A. carbonarius*	+
A.C2	*A. carbonarius*	+
A.C5	*A. carbonarius*	+
A.C6	*A. carbonarius*	+
A.C7	*A. carbonarius*	+
A.C8	*A. carbonarius*	+
A.C9	*A. carbonarius*	+
A.C11	*A. carbonarius*	+
A.C12	*A. carbonarius*	+

Table 6 Inclusivity test results for the primer set AFS03

Strain	Species	Result
BP50	*A. flavus*	+
BP51	*A. flavus*	+
BP53	*A. flavus*	+
AS6	*A. flavus*	+
AS3	*A. flavus*	+
AS9	*A. flavus*	+
AS15	*A. flavus*	+
AS10	*A. flavus*	+
AS8	*A. flavus*	+
AS7	*A. flavus*	+
Aca10	*A. flavus*	+

A.F5	*A. flavus*	+
A.F9	*A. flavus*	+

Table 7 Exclusivity test results for the primer set ACS02

Strain	Species	Result	Strain	Species	Result	Strain	Species	Result
BP37	*A. niger*	-	BP51	*A. niger*	-	AS6	*A. flavus*	-
BP8	*A. niger*	-	ACa1	*A. niger*	-	AS3	*A. flavus*	-
BP4	*A. niger*	-	BP29	*A. tubingensis*	-	AS9	*A. flavus*	-
BP10	*A. niger*	-	BP33	*A. tubingensis*	-	AS15	*A. flavus*	-
BP16	*A. niger*	-	BP41	*A. tubingensis*	-	AS10	*A. flavus*	-
BP18	*A. niger*	-	BP43	*A. tubingensis*	-	AS8	*A. flavus*	-
BP26	*A. niger*	-	BP50	*A. flavus*	-	AS7	*A. flavus*	-
BP27	*A. niger*	-	BP48	*A. flavus*	-	BP34	*A. niger*	-
BP30	*A. niger*	-	BP53	*A. flavus*	-	BP35	*A. niger*	-
BP31	*A. niger*	-	AS17	*A. tamarii*	-	A.Ca10	A. flavus	-

Table 8 Exclusivity test results for the primer set AFS03

Strain	Species	Result
PB4	*A. niger*	-
PB8	*A. niger*	-
PB10	*A. niger*	-
PB16	*A. niger*	-
A.P1	*A. parasiticus*	-
AS17	*A. tamarii*	-
BP29	*A. tubingensis*	-
BP33	*A. tubingensis*	-
A1P1	*Penicillium sp.*	-
A1P5	*Penicillium sp.*	-
A1P6	*Penicillium sp.*	-
A1P8	*Penicillium sp.*	-
A1P13	*Penicillium sp.*	-
A1P1	*Penicillium sp.*	-
BP36	*A. carbonarius*	-
A.C1	*A. carbonarius*	-
A.C2	*A. carbonarius*	-

\+ Isolate detected by the LAMP assay (positive result)

\- Isolate non detected by the LAMP assay (negative result)
